# Comparison of clinical features, management and outcomes of osteosarcoma located in proximal fibula and proximal tibia: a propensity score matching analysis

**DOI:** 10.1186/s12885-018-5062-6

**Published:** 2018-11-29

**Authors:** Hao Yao, Bo Wang, Lili Wen, Qinglin Jin, Hongbo Li, Gang Huang, Junqiang Yin, Changye Zou, Xianbiao Xie, Jingnan Shen

**Affiliations:** 1 Department of Musculoskeletal Oncology Center, The First Affiliated Hospital of Sun Yat-sen University, 58 Zhongshan 2nd Rd, Guangzhou, 510080 China; 2 Department of Anesthesiology, State Key Laboratory of Oncology in South China, Sun Yat-sen University Cancer Center, 651 Dongfengdong Rd, Guangzhou, 510060 China

**Keywords:** Osteosarcoma, Proximal fibula, Proximal tibia, Amputation rates, Clinical outcomes

## Abstract

**Background:**

The aim of this study was to compare proximal fibular and proximal tibial sites regarding osteosarcoma in the proximal crus. Furthermore, we proposed a hypothesis explaining the differences.

**Methods:**

From Jaunary 2000 to February 2015, 28 patients with non-metastatic proximal fibular osteosarcoma and 214 patients with non-metastatic proximal tibial osteosarcoma underwent surgery were identified in our center. All clinical data were analyzed retrospectively. Propensity score matching of patients in a 1:2 ratio was conducted based on age, gender and Enneking stage. To analyze possible factors resulting in amputation, we investigated extraosseous tumor volumes (ETVS), the nearest of the blood vessel to reactive zone (NBR) and the nearest of the blood vessel to tumor (NBT).

**Results:**

Amputation rates were higher in the proximal fibula cohort (35.7%) than in the proximal tibia cohort (14.3%; *p* = 0.046). Comparing possible clinical characteristics related with amputation between two cohorts, the proximal fibula cohort had larger ETVS (*p* = 0.000). Moreover, the proximal fibula cohort had a smaller NBT for anterior tibial vessels (*p* = 0.025), a smaller NBR for posterior tibial vessels (*p* = 0.013) and a smaller NBT for posterior tibial vessels (*p* = 0.007) than the proximal tibia cohort. Univarite and multivariable analyses showed that NBT for posterior tibial vessels was the only independent factor associated with amputation. The 3-year event-free survival (EFS) and overall survival (OS) rates for the proximal fibula cohort vs. the proximal tibia cohort were 52.6% vs. 78.0% (*p* = 0.045) and 63.7% vs. 81.2% (*p* = 0.177), respectively. The MSTS scores for the functional evaluation of limb-salvaging surgery were similar in both groups (*p* = 0.212).

**Conclusions:**

Amputation rates among patients were higher when osteosarcoma was located in proximal fibula than in proximal tibia. A smaller NBT for posterior tibial vessels was associated with higher amputation rates. Prognosis of the proximal fibula cohort was poorer than that of the proximal tibia cohort of osteosarcoma patients.

**Electronic supplementary material:**

The online version of this article (10.1186/s12885-018-5062-6) contains supplementary material, which is available to authorized users.

## Background

Osteosarcoma is the most common primary malignant bone tumor in children and young adults [1]. Distal femur, proximal tibia and proximal humerus are recognized as the three most frequent lesion sites. In addition, more than 70% osteosarcoma originates around the knee joint [2]. Surgery including amputation and limb-salvaging procedures remains an essential part of management [3], and the addition of neoadjuvant and adjuvant chemotherapy to surgery has improved the outcomes. As currently reported, the 5-year overall survival (OS) of patients with localized osteosarcoma is approximately 70% [4–6].

As a bony structure near the knee joint, the proximal fibula is a rare site of osteosarcoma. As reported, only 2% of primary osteosarcoma is located in the fibula, and this proportion is lower in the proximal fibula [7]. The proximal fibula and proximal tibia are two adjacent structures in proximal crus; thus, in addition to the difference in incidence rate, the other characteristics of osteosarcoma lesions at these two sites may not be similar according to our clinical experience.

The fibula is recognized as a small and expandable bone without irreplaceable function for bearing body weight and movement, among others. In contrast, the tibia is a large bone attached to several functional muscles, tendons and ligaments and is a component of the knee joint, which acts as an indispensable structure for bearing body weight and movement of lower limbs. Limb-salvaging procedure for proximal fibula seems to be simpler than that for proximal tibia, because reconstruction after resection is not essential. The long-term complications of artificial prosthesis are also a matter of concern.

Regarding clinical outcomes, Sean P et al. compared the 5-year OS of fibular osteosarcoma patients in their study with results reported by other investigators and observed similar outcomes. However, this result may not be true when the tumor located in the proximal part of the fibula [8]. Kutikov et al. reported that osteosarcoma in proximal fibula may easily invade two or all three compartments of the proximal crus [9], and Seker et al. observed a higher likelihood of inadequate surgical margins in osteosarcoma in fibula than in other sites for patients undergoing limb-salvaging surgery [10].

Nevertheless, perhaps because of the low incidence of osteosarcoma in proximal fibula, there is a lack of previously published study on the topic. The difference between osteosarcoma in proximal fibula and osteosarcoma in proximal tibia still needs to be clarified. Therefore, the comparison of osteosarcoma originating in these two sites is an attractive topic of research.

The purpose of the present study was to compare the clinical features, management and outcomes of osteosarcoma located in proximal fibula and proximal tibia. Furthermore, we proposed a possible hypothesis to account for the differences.

## Methods

### Study population

We retrospectively reviewed the case records of 1133 patients with a histologic diagnosis of osteosarcoma during the period of January 2000 to February 2015 at the First Affiliated Hospital of Sun Yat-sen University.

The inclusion criteria were as follows: (1) patients with histologically confirmed conventional high-grade osteosarcoma at the First Affiliated Hospital of Sun Yat-sen University; (2) tumors located in proximal fibula or proximal tibia; (3) patients who did not receive any antitumor therapy before admission in our center; (4) patients underwent magnetic resonance imaging (MRI) at initial presentation; and (5) complete clinical and follow-up data were available. The exclusion criteria were as follows: (1) evidence of distal metastasis at initial diagnosis and; (2) no experience of receiving standard treatment protocol in our center including neoadjuvant, surgery and adjuvant chemotherapy.

Followed the inclusion criteria, we initially identified 39 (3.4%) patients with proximal fibular osteosarcoma. Eleven cases were excluded because of distal metastasis at diagnosis (5 patients) or lack of surgery (6 patients). Eventually, we included 28 patients in the proximal fibula for matching. At the same time, we identified 214 (18.9%) patients with proximal tibial osteosarcoma. Among them, 68 cases were excluded because of distal metastasis at diagnosis (25 patients) or lack of surgery (43 patients). Eventually, we included 146 patients in the proximal tibia for matching.The flow diagram for inclusion is presented in Additional files.

### Treatment strategy

A core needle biopsy was performed for definitive diagnosis in every patient. The tumor-invaded extremity was evaluated by plain X-ray and MRI before any treatment was performed. A computed tomography (CT) scan of the lung and whole body emission computed tomography (ECT) scan were employed to exclude distal metastasis and to confirm Enneking staging [11].

A total of four commonly used chemotherapeutic agents were administered according to the scheme presented in Additional file 1: Figure S1 Methotrexate (MTX), cisplatin (DDP), doxorubicin(ADM) and ifosfamide(IFO). The interval between rounds of chemotherapy was 2 to 3 weeks. Then, 2 or 3 weeks after surgery, the patients would receive adjuvant chemotherapy if no complications were noted. Patients in both cohorts received standard chemotherapy consisting of 4 neo-adjuvant chemotherapy courses and 9 or more adjuvant chemotherapy courses. Good response to neo-adjuvant chemotherapy was assessed in terms of (1) relief of pain, (2) hardening or diminution of local mass, (3) decreased serum alkaline phosphatase (ALP) level for osteosarcoma, and (4) decreased mass volume or clearer tumor border with less soft tissue edema, according to MR scan.

Limb-salvage surgery was more likely to be performed if possible in our center. Contraindications for limb-salvaging surgery were as follows: (1) en-bloc resection is extremely difficult; (2) unacceptable limb function after surgery; (3) bad response or poor tolerance to chemotherapy; (4) displaced pathological fracture; and (5) lack of willingness of patients and family members. Suggestions were provided after carefully evaluating the individual situation by discussion with bone tumor specialists.

For patients with proximal fibular osteosarcoma, limb-salvaging surgery was performed following the Malawer type-II wide resection procedure, in which the common peroneal nerve was sacrificed [12]. For patients with proximal tibial osteosarcoma, wide resection of the tumor, reconstruction of the bone and joint defect by artificial prosthesis, reconstruction of the soft tissue by transfer of medial head of medial head of gastrocnemius were performed.

### MRI assessment

The differentiation of extraosseous tumor from reactive zone was determined followed Philipp Lang’s criteria [13].

Tumor volumes (TVS) were calculated assuming an ellipsoidal configuration using Gobel’s method [14]: TVS = π × (tumor length) × (tumor width) × (tumor depth)/6. Extraosseous tumor volumes (ETVS) were defined as the difference between TVS and the volumes of the intraosseous part of the tumor. ETVS were also determined assuming as an ellipsoidal configuration: ETVS = π × (tumor length) × ((tumor width) × (tumor depth)-(intraosseous tumor width) × (intraosseous tumor depth))/6.

**Fig. 1:**
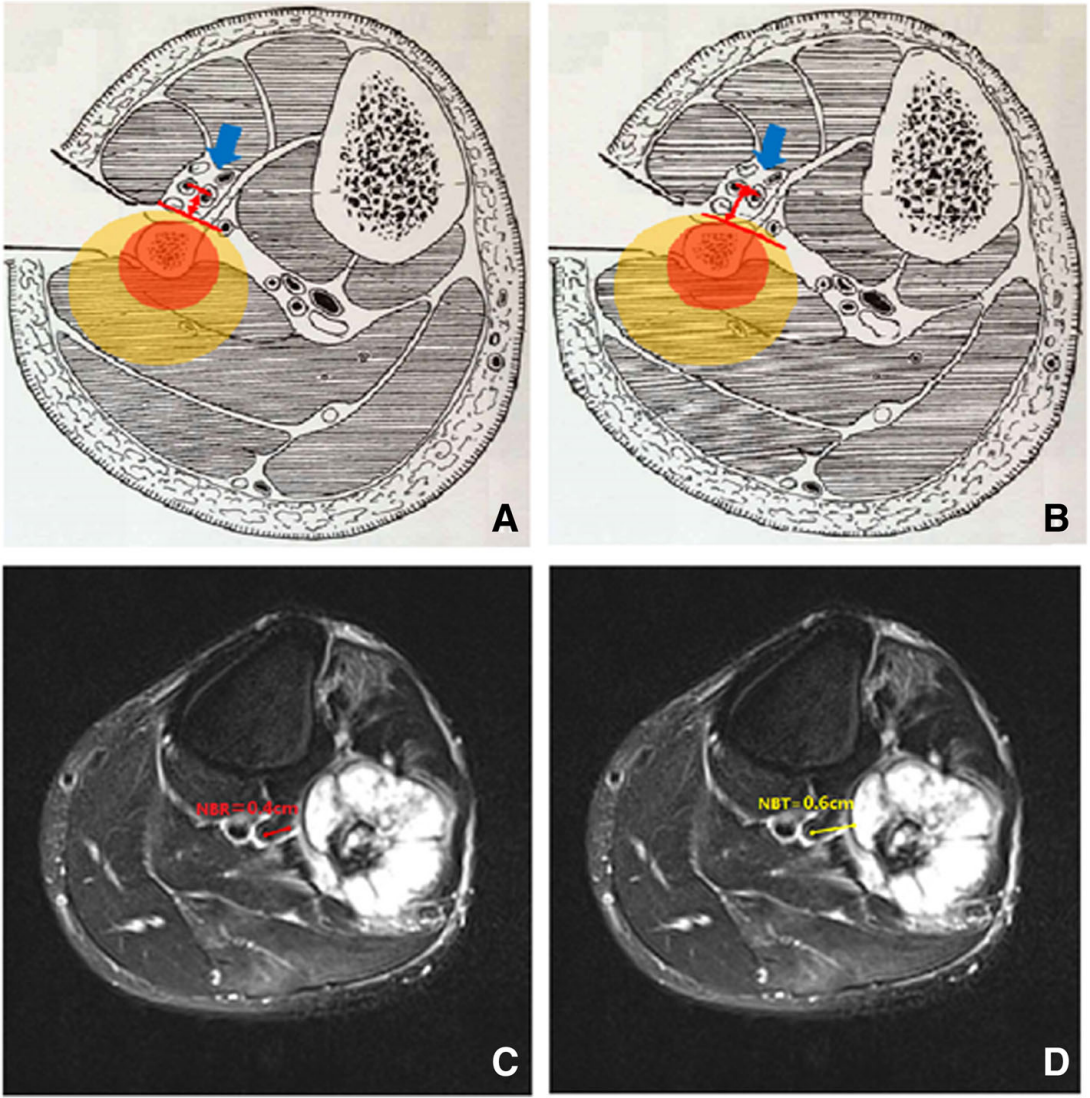
Measurement of nearest of the blood vessels to reactive zone (NBR) and nearest of the blood vessels to tumor (NBT). **a** Diagram of NBR of politeal vessels. **b** Diagram of NBT of politeal vessels. **c** NBR of popliteal vessels was 0.4 cm. **d** NBT of popliteal vessel was 0.46 cm

Three main blood vessels including the main branch of popliteal, anterior tibial and posterior tibial vessels were evaluated in our study. Referring to the idea of design in R.E.N.A.L. nephrometry scoring system [15], we used the following two definitions to quantify the relationship between blood vessels and lesions: (1) the nearest of blood vessel to the reactive zone (NBR): the shortest distance between the center of main branch of the blood vessels and the margin of the reactive zone; (2) the nearest of blood vessel to the tumor (NBT): the shortest distance between the center of main branch of the blood vessels and the tumor margin (Fig. 1). We estimated the values of NBR and NBT under the following circumstances: (1) NBR was noted as 0.01 cm when blood vessels were adjacent to the margin of reactive zone; (2) NBR was noted as 0 cm when blood vessels were in the reactive zone; (3) NBT was noted as 0.01 cm when blood vessels were adjacent to the margin of tumor; and (4) NBR was noted as 0 cm when blood vessels were surrounded by the tumor.

The measurement procedure was performed independently by two medical imaging specialists, and discussions were carried out when disagreements occurred until consensus was achieved.

### Follow-up

Regular follow-up was scheduled every 3 months in the first 2 postoperative years, every 6 months for the following 3 years, and yearly thereafter [16]. The latest follow-up was February 2018. The definition of event-free survival (EFS) was the time from definitive diagnosis to the time of disease progression, recurrence, second primary malignancy, or death due to any cause or the last follow-up.

Functional evaluation for patients underwent limb-salvaging surgery was done at 6 months after surgery using the Musculoskeletal Tumor Society Scoring System 93 (MSTS 93) criteria by Enneking et al. The items included pain, function, emotional acceptance, support, walking and gait.

### Statistical methods

Statistical analyses were performed using SPSS Statistics for Windows (version 24.0; IBM). Associations between categorical variables were studied using the Pearson’s chi-square test if the sample size was greater than 5 and using Fisher’s exact test if the sample size was less than 5. For continuous variables, we used Student t tests. Logistic regression analysis was conducted to estimate the Odds ratios for factors resulted in amputation. OS and EFS distributions were estimated by using the Kaplan Meier method, and the subgroup survival analysis was performed using the log-rank test. The level of significance was set at< 0.05.

Variables with difference between groups might result in misleading outcomes. Potential covariables included in propensity score matching were age, gender and Enneking stage. Propensity scores were estimated using a logistic regression model. A 1-to-2 nearest neighbor matching algorithm was applied with a caliper of 0.2 and without replacement. Standardized mean differences and linear plot of individual differences were shown in order to examine the outcome of propensity score matching.

## Results

### Patient’s baseline demographics before and after propensity score matching

Baseline characteristics of 28 proximal fibular and 146 proximal tibial osteosarcoma patients are summarized in Table 1. Compared with the proximal fibula group, the 146 proximal tibia group had the following numerical difference: younger age, more female and more Enneking IIB stage.

**Table 1:** Summary of patients’ demographics and tumor characteristics before propensity score matching

	Proximal Fibula (*N* = 28)	Proximal Tibia (*N* = 146)	*P* Value^*^	Standardized Difference
Mean age at time of diagnosis (yr)	18.6 ± 7.8	18.0 ± 8.7	0.746	0.069
Sex			0.178	0.386
Female	5 (17.9%)	46 (31.5%)		
Male	23 (82.1%)	100 (68.5%)		
Enneking Stage			0.639	0.090
IIA	2 (7.1%)	7 (4.8%)		
IIB	26(92.9%)	139 (95.2%)		

*Abbreviations*: *yr.* year, *mo* month

^*^The p value was calculated using the Fisher’s exact test for categorical variables and t-test for continuous variables

**Fig. 2:**
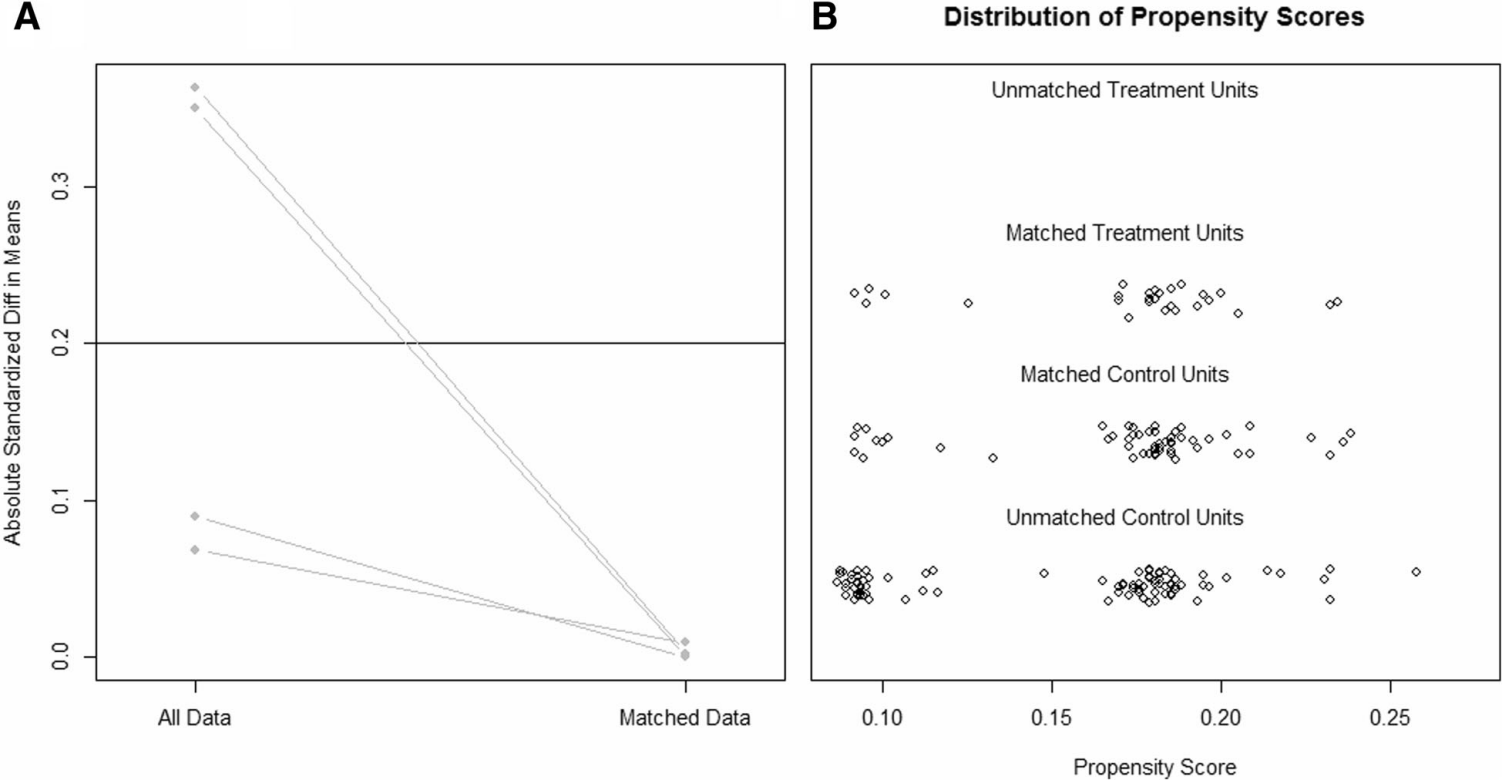
Line plots of standardized differences of this study before and after propensity score matching. **a** Parallel line plot of the standardized difference in means before and after PSM. **b** Dot plot of the propensity scores of patients in proximal fibula group and proximal tibia group

After propensity score matching, we compared the characteristics of the 28 patients selected from the proximal fibula group (the matched control group) and the other 56 patients selected from the proximal tibia group (the matched case group) (Table 2). The absolute standardized difference decreased from 0.363 to 0.004. The propensity score in matched groups was evenly distributed. (Fig. 2).

**Table 2:** Patients’ demographics and tumor characteristics of the two matched cohorts after propensity score matching

	Proximal Fibula (*N* = 28)	Proximal Tibia (*N* = 56)	*P* Value^*^	Standardized Difference
Mean age at time of diagnosis (yr)	18.6 ± 7.8	18.5 ± 6.6	0.965	0.011
Sex			1.000	0.000
Female	5 (17.9%)	10 (17.9%)		
Male	23 (82.1%)	46 (82.1%)		
Enneking Stage			1.000	0.000
IIA	2 (7.1%)	4 (7.1%)		
IIB	26 (92.9%)	52 (92.9%)		

*The *p* value was calculated using the Fisher’s exact test for categorical variables and t-test for continuous variables

### Comparision of clinical characteristics related with amputation

Ten patients in the proximal fibula cohort and 8 patients in the proximal tibia cohort underwent amputation. The amputation rate in the proximal fibula cohort was significantly higher than that in the proximal tibia cohort (35.7% vs. 14.3%; *p* = 0.046).

Possible clinical characteristics related with amputation were compared between proximal fibula cohort and proximal tibia cohort (Table 3).The time from the appearance of symptom to diagnosis was similar in both cohorts (mean, 2.95 months vs. 3.02 months; *p* = 0.901). The mean TVS were similar in cohorts, 98.21 cm^3^ in the proximal fibula cohort and 124.25 cm^3^ in the proximal tibia cohort (*p* = 0.141). ETVS were significantly larger in the proximal fibula cohort than in the proximal tibia cohort (mean; 83.92 cm^3^vs.31.64 cm^3^; *p* = 0.000). No significant difference was found in NBT and NBR regarding popliteal vessels between cohorts. The proximal fibula cohort had a smaller NBT for anterior tibial vessels than the proximal tibia cohort (mean, 0.09 cm vs. 0.80 cm; *p* = 0.025). The NBR for posterior tibial vessels in the proximal fibula cohort was smaller than that in the proximal tibia cohort (mean, 0.06 cm vs. 0.46 cm; *p* = 0.013). The NBT for posterior tibial vessels in the proximal fibula cohort was also smaller than that in the proximal tibia cohort (mean, 0.56 cm vs. 1.42 cm; *p* = 0.017). Moreover, NBR of anterior tibial vessels was smaller in the proximal fibula cohort than in the proximal tibia cohort, although not significantly different.

**Table 3:** Comparision of clinical characteristics related with amputation

Variable	Prxoximal Fibula (*N* = 28)	Proximal Tibia (*N* = 56)	*P* Value^*^
Mean time between symptom appeared to diagnosis (mo)	2.95	3.02	0.901
Tumor volumes (cm^3^)	98.21	124.52	0.141
Extraosseous Tumor Volumes (cm^3^)	83.92	31.64	0.000
Nearest to popliteal vessels (mean; cm)			
Reactive zone	0.18	0.27	0.537
Tumor	0.63	1.39	0.106
Nearest to anterior tibial vessels (mean; cm)			
Reactive zone	0.01	0.16	0.199
Tumor	0.09	0.80	0.025
Nearest to posterior tibial vessels (mean; cm)			
Reactive zone	0.06	0.46	0.013
Tumor	0.56	1.42	0.017

Abbreviations: mo month

*All *p* values were calculated using t-test for continuous variables

### Analysis of possible factors resulted in amputation

Univariate analysis revealed that NBT for politeal vessels (*p* = 0.016), NBT for anterior tibial vessels (*p* = 0.020), NBR for posterior tibial vessels (*p* = 0.008) and NBT for posterior tibial vessels (*p* = 0.010) had a significant association with amputation (Table 4). A multivariable analysis that was conducted with clinical variables related to amputation revealed that NBT for posterior tibial vessels was the only independent factor for amputation (*p* = 0.026).

**Table 4:** Univariate analysis for possible factors resulted in amputation

Variable	Amputation (*N* = 18)	Limb Salvage (*N* = 66)	*P* Value^*^
Mean time between symptom appeared to diagnosis (mo)	2.89 ± 1.83	2.96 ± 2.63	0.947
Tumor volumes (cm^3^)	131.06 ± 86.46	96.70 ± 75.21	0.343
Extraosseous Tumor Volumes (cm^3^)	83.57 ± 78.75	39.09 ± 34.65	0.144
Nearest to popliteal vessels (mean; cm)			
Reactive zone	0.05 ± 0.15	0.31 ± 0.39	0.052
Tumor	0.45 ± 0.51	1.33 ± 0.90	0.016
Nearest to anterior tibial vessels (mean; cm)			
Reactive zone	0.00 ± 0.00	0.11 ± 0.27	0.192
Tumor	0.08 ± 0.25	0.62 ± 0.65	0.020
Nearest to posterior tibial vessels (mean; cm)			
Reactive zone	0.00 ± 0.00	0.36 ± 0.38	0.008
Tumor	0.41 ± 0.56	1.35 ± 0.84	0.010

*The *p* value was calculated using the Fisher’s exact test for categorical variables and t-test for continuous variables

### Comparison of event-free survival and overall survival

**Fig. 3:**
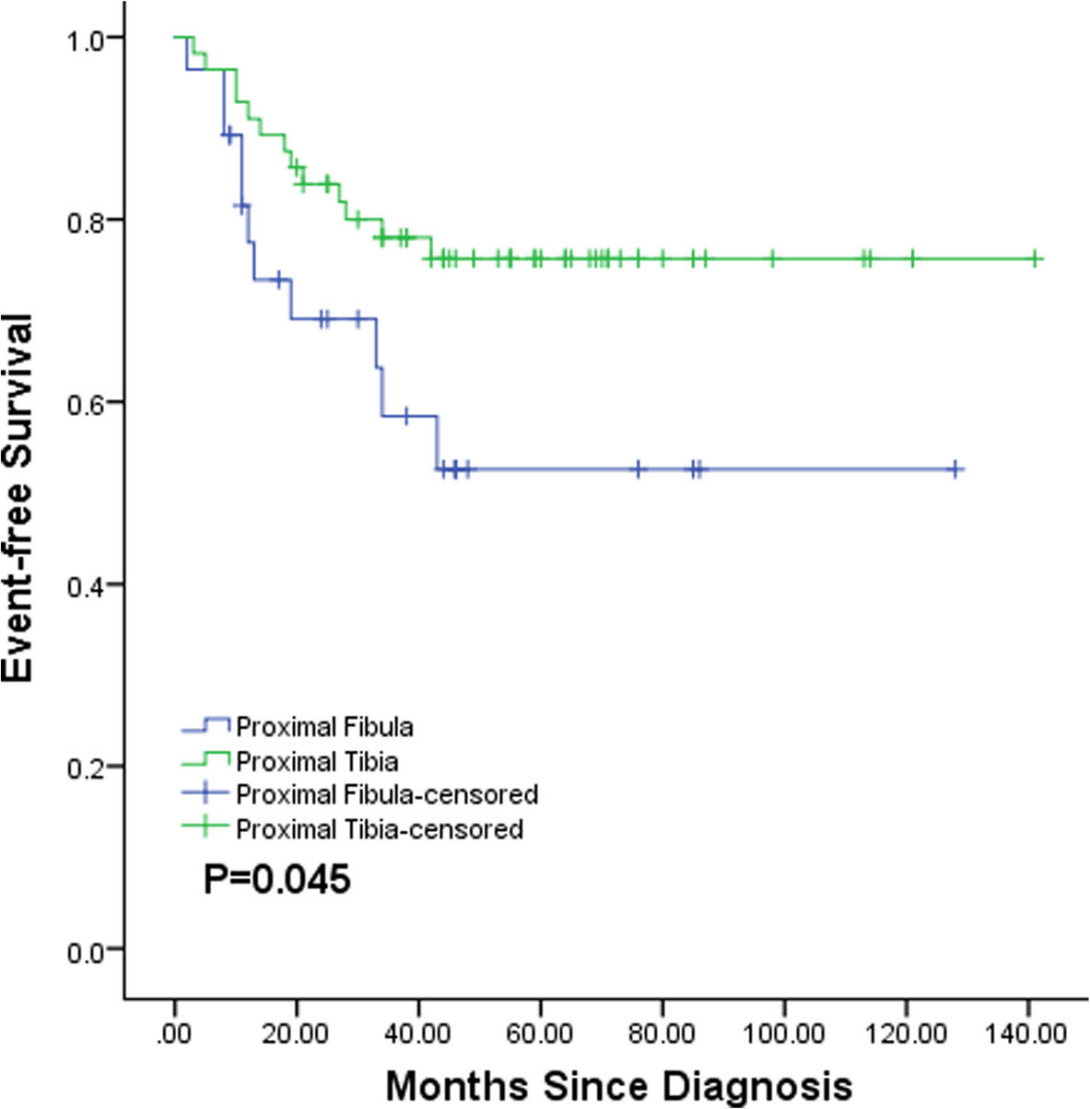
Event-free survival of proximal fibular (*N* = 28) and proximal tibial (*N* = 56) osteosarcoma patients

With a median follow-up of 44.5 months (range 6–141 months), events occurred in 11 patients (39.3%) in the proximal fibula cohort and in 13 patients (23.2%) in the proximal tibia cohort. The 3-year cumulative EFS rates were 52.6 and 78.0%, respectively, for the proximal fibula cohort and proximal tibia cohort. The proximal fibula cohort had poorer EFS than the proximal tibia cohort (*p* = 0.045) (Fig. 3).

**Fig. 4:**
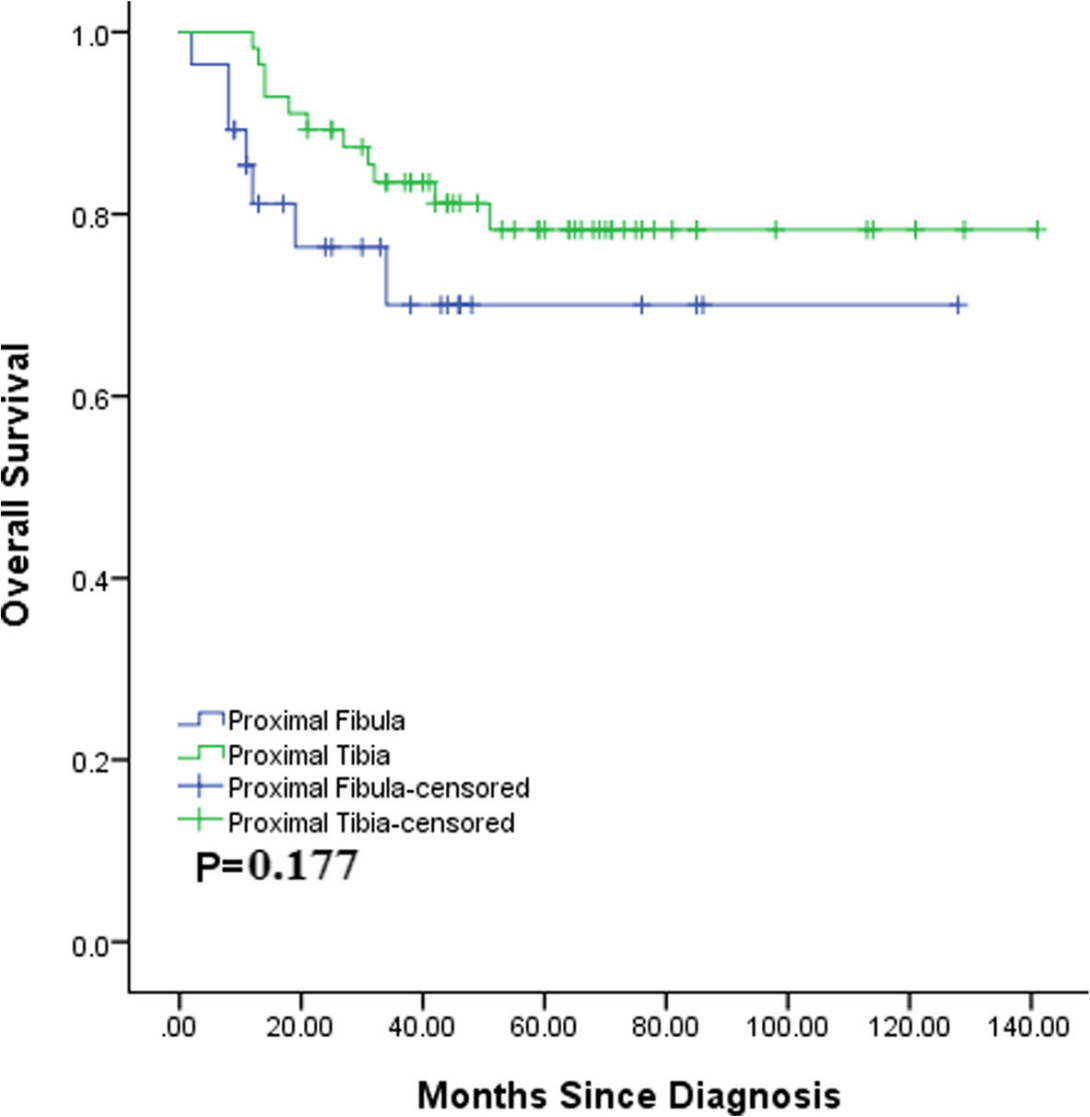
Overall survival of proximal fibular (*N* = 28) and proximal tibial (*N* = 56) osteosarcoma patients

During the follow-up time, we noted 7 deaths (25.0%) in the proximal fibula cohort and 11 deaths (19.6%) in the proximal tibia cohort. The 3-year OS rates for the proximal fibula and proximal tibia cohorts were 63.7 and 81.2%, respectively. There was no significant difference between the two matched cohorts (*p* = 0.177) (Fig. 4).

### Comparison of functional evaluation

For the 18 patients in the proximal fibula cohort and 48 patients in the proximal tibia cohort who underwent limb-salvaging surgery, functional evaluations were performed using the MSTS 93at 6 months after surgery. Generally, the mean of sum scores was similar in the proximal fibula cohort and the proximal tibia cohort (20.1 ± 2.3 vs. 21.2 ± 3.4; *p* = 0.212). Listed separately, the proximal fibula cohort had better scores for emotional acceptance than the proximal tibia cohort (mean; 3.7 vs. 3.1; *p* = 0.016). However, the proximal fibula cohort had poorer scores than the proximal tibia cohort in support (mean; 2.9 vs. 3.5; *p* = 0.003) and walking (mean; 3.0 vs. 3.6; *p* = 0.003). The score of pain, function and gait were similar in both cohorts without significant difference. Table 5 shows the results of functional evaluation based on MSTS 93.

**Table 5:** Functional evaluation based on the musculoskeletal tumor society scoring system (MSTS 93)

	5 points (Fibula/Tibia)	3–4 points (Fibula/Tibia)	1–2 points (Fibula/Tibia)	0 points (Fibula/Tibia)	Means (Fibula/Tibia)
Pain	10/32	8/16	0/0	0/0	4.5 ± 0.6/4.5 ± 0.7
Function	0/7	15/39	3/2	0/0	3.1 ± 0.7/3.5 ± 0.8
Emotional Acceptance	4/0	11/41	3/7	0/0	3.7 ± 0.9/3.1 ± 0.7
Supports	0/14	16/21	2/13	0/0	2.9 ± 0.3/3.5 ± 1.2
Walking	0/3	14/44	4/1	0/0	3.0 ± 0.7/3.6 ± 0.6
Gait	0/1	16/39	2/8	0/0	2.9 ± 0.3/3.1 ± 0.7

## Discussion

From January 2000 to February 2015, we identified 1133 patients with a histologic diagnosis of osteosarcoma. The incidence of osteosarcoma in the proximal fibula was 3.4%, and that in the proximal tibia was 18.9%. The incidence of proximal fibular osteosarcoma in our study was low and similar to that reported by Ozaki et al. [7].

The amputation rates of osteosarcoma located in proximal fibula (35.7%) is not only higher than that of osteosarcoma located in proximal tibia (14.3%) based on our findings, but also higher than the average amputation rates in extremities [17]. As previously reported in several studies, with preoperative neoadjuvant chemotherapy, fewer than 20% patients with nonmetastatic osteosarcoma of the extremity finally underwent amputation [18–21]. Therefore, the identification of possible factors resulting in high amputation rates of proximal fibular osteosarcoma is naturally the following topic of investigation.

We noted larger ETVS in the proximal fibula cohort (83.92cm^3^) than in the proximal tibia cohort (30.33 cm^3^). Because of the large difference in volumes of tumors in proximal fibula and proximal fibula, ETVS might thus better represent the degree of invasion of surrounding structures of the tumor than TVS. The proximal fibula cohort had a smaller value of NBR for the main branch of anterior tibial vessels than did the proximal tibia cohort, indicating a closer relationship between anterior tibial vessels and the tumor. Moreover, the proximal fibula cohort had a smaller value of NBT for the main branch of posterior tibial vessels than did the proximal tibia cohort, indicating a closer relationship between posterior tibial vessels and tumor. Invasion of tumors into main blood vessels is an obstacle for limb-salvaging operation. Because adequate surgical margin can be difficult to achieve if the invaded blood vessels are not sacrificed, but it is impossible to resect anterior tibial vessels and posterior tibial vessels at the same time or the crus will not actually exist. Hence, the relationship between anterior tibial vessels and tumors and between posterior tibial vessels and tumors in osteosarcoma may be responsible for amputation. Given the anatomical structure, the cortical bone of the proximal fibula is thinner than that of the proximal tibia; thus, the tumor may more easily break through the cortex and invade the soft tissue. Another concern for proximal tibia is that posterior tibial vessels and tibial nerve are protected by tibialis and popliteus, and these muscles may also act as an obstacle for invading tumors. Furthermore, anterior tibial vessels, deep peroneal nerve and common peroneal nerve surround the neck of the fibula, and these structures may be easily invaded by the tumor.

Based on our results, the EFS in the proximal fibula cohort was poorer than that in the proximal tibia cohort. The OS in the proximal fibula cohort was also poorer, albeit not significant. Previously, some investigators mentioned tumor site as a prognostic factor for osteosarcoma [22–24]. For instance, as commonly accepted, extremity tumors were thought to confer better survival rates than non-extremity tumors [22–24]. And as Bidlack et al. reported that tumors in proximal tibia resulted in better prognosis than tumors in femur or humerus [22]. The included cases in both cohorts of our study had similar age, sex, surgical and stage and underwent standard treatment, but prognosis in proximal fibula was better than that in proximal tibia. Metastasis of osteosarcoma is mostly thought to occur via blood vessels. When tumors and main blood vessels are closely associated, the chance of metastasis may be higher. Furthermore, metastasis is an important prognostic factor of osteosarcoma, and thus, it is reasonable to suspect that invasion into main blood vessels may also be a prognostic factor in osteosarcoma. However, due to the emphasis of our study, this hypothesis needs to be explored further in future studies.

In the functional evaluation at 6 months after surgery for limb-salvaging patients, the sum of scores was similar and acceptable in both cohorts. Nevertheless, the better emotional acceptance score for the proximal fibula cohort than in the proximal tibia cohort may be due to limited surgical trauma and simple recovery procedure. In our center, Malawer type-II en-bloc resection with sacrifice of the peroneal nerve was routinely performed in patients with proximal fibular osteosarcoma; thus, ankle-foot orthosis was essential. As a result, the score for support may be worse in the proximal fibula cohort than in the proximal tibia cohort. We think that in the proximal tibial osteosarcoma patients, the long-term complications of artificial prosthesis may be a potential matter of concern. Due to the limited interval between surgery and functional evaluation in our study, the result of comparison of function between groups still need to be clarified in further follow-up.

It should be acknowledged that our conclusion may be influenced by the limited number of cases or by the confounding factors during matching. The reason for the higher amputation rates and poorer prognosis in the fibula cohort than in the tibia cohort needs to be cautiously interpreted and validated by other studies with larger sample sizes.

## Conclusions

Amputation rates among patients with osteosarcoma were higher when tumors were located in the proximal fibula than in the proximal tibia. The factors resulting in higher amputation rates may be the closer relationship between posterior tibial vessels and tumor. Moreover, EFS of patients with osteosarcoma in the proximal fibula was poorer than that of patients with osteosarcoma in the proximal tibia. The results of functional evaluation at 6 months after limb-salvaging surgery were similar in both groups.

## Additional file

Additional file 1: **Figure S1.** The flow diagram for inclusion of patients. This flow diagram shows the process of inclusion of patients. **Figure S2.** An example of chemotherapy protocol. This picture shows an example of chemotherapy protocol with 9 courses of adjuvant chemotherapy, patients included in our study received 9 or more adjuvant chemotherapy courses. **Figure S3.** An example of measuring tumor volumes (TVS) and extraosseous tumor volumes (ETVS). A. Measurement of tumor length from coronary section. B. Measurement of tumor width and depth from transverse section. C. Measurement of intraosseous tumor length from coronary section. D. Measurement of intraosseous tumor width and depth from transverse section. (DOCX 531 kb)

## Abbreviations

CT: Computed tomography
ECT: Emission computed tomography
EFS: Event-free survival
ETVS: Extraosseous tumor volumes
MRI: Magnetic resonance imaging
MSTS 93: Musculoskeletal Tumor Society Scoring System 93
NBR: The nearest of blood vessel to the reactive zone
NBT: The nearest of blood vessel to the tumor
OS: Overall survival
TVS: Tumor volumes

## References

[1] Mirabello L, Troisi RJ, Savage SA (2009). Osteosarcoma Incidence and Survival rates from 1973 to 2004: data from the surveillance, epidemiology, and End Results Program. Cancer Am Cancer Soc.

[2] Ottaviani G, Jaffe N (2009). The epidemiology of osteosarcoma. Cancer Treat Res.

[3] Marulanda GA, Henderson ER, Johnson DA, Letson GD, Cheong D. Orthopedic surgery options for the treatment of primary osteosarcoma. Cancer Control. 2008;(1):13–20. 10.1177/107327480801500103 18094657

[4] Ferrari S, Smeland S, Mercuri M, et al. Neoadjuvant chemotherapy with high-dose Ifosfamide, high-dose methotrexate, cisplatin, and doxorubicin for patients with localized osteosarcoma of the extremity: a joint study by the Italian and Scandinavian sarcoma groups. J Clin Oncol. 2005;(34):8845–52. 10.1200/JCO.2004.00.5785 16246977

[5] Eilber F, Giuliano A, Eckardt J, Patterson K, Moseley S, Goodnight J. Adjuvant chemotherapy for osteosarcoma: a randomized prospective trial. J Clin Oncol. 1987;(1):21–6. 10.1200/JCO.1987.5.1.21 3543236

[6] Meyers PA, Schwartz CL, Krailo M (2005). Osteosarcoma: a randomized, prospective trial of the addition of Ifosfamide and/or Muramyl tripeptide to cisplatin, doxorubicin, and high-dose methotrexate. J Clin Oncol.

[7] Ozaki T, Hillmann A, Lindner N, Winkelmann W (1997). Surgical treatment of bone sarcomas of the fibula. Analysis of 19 cases. Arch Orthop Trauma Surg.

[8] Schneiderbauer MM, Gullerud R, Harmsen WS, Scully SP (2007). Fibular osteosarcomas: contaminated margins may not impact survival. Clin Orthop Relat Res.

[9] Yan TQ, Yang RL, Guo W (2008). The clinical outcome of proximal fibular osteosarcoma with En-bloc resection. Zhonghua wai ke za zhi [Chinese journal of surgery].

[10] Grimer RJ, Taminiau AM, Cannon SR. Surgical Outcomes in Osteosarcoma. J Bone Joint Surg Br. 2002;(3):395–400. 10.1302/0301-620x.84b3.12019 12002500

[11] Enneking WF (1986). A system of staging musculoskeletal neoplasms. Clin Orthopaed Relat Res.

[12] Malawer MM (1984). Surgical Management of Aggressive and Malignant Tumors of the proximal fibula. Clin Orthop Relat Res.

[13] Lang P, Honda G, Roberts T, et al. Musculoskeletal neoplasm: Perineoplastic edema versus tumor on dynamic Postcontrast MR images with spatial mapping of instantaneous enhancement rates. Radiology. 1995;(3):831. 10.1148/radiology.197.3.7480764 7480764

[14] Göbel V, Jürgens H, Etspüler G (1987). Prognostic significance of tumor volume in localized Ewing’s sarcoma of bone in children and adolescents. A system of staging musculoskeletal neoplasms. J Cancer Res Clin Oncol.

[15] Kutikov A, Uzzo RG (2009). The R.E.N.a.L. Nephrometry score: a comprehensive standardized system for quantitating renal tumor size, location and depth. J Urol.

[16] Rothermundt C, Seddon BM, Dileo P (2016). Follow-up practices for high-grade extremity osteosarcoma. BMC Cancer.

[17] Rougraff BT, Simon MA, Kneisl JS, Greenberg DB, Mankin HJ (1994). Limb salvage compared with amputation for osteosarcoma of the distal end of the femur. A long-term oncological, functional, and quality-of-life study. J Bone Joint Surg Am.

[18] Bacci G, Ferrari S, Bertoni F (2000). Long-term outcome for patients with nonmetastatic osteosarcoma of the extremity treated at the Istituto Ortopedico Rizzoli according to the Istituto Ortopedico Rizzoli/Osteosarcoma-2 protocol: an updated report. J Clin Oncol.

[19] Bacci G, Picci P, Ferrari S (1993). Primary chemotherapy and delayed surgery for nonmetastatic osteosarcoma of the extremities. Results in 164 patients preoperatively treated with high doses of methotrexate followed by cisplatin and doxorubicin. Cancer Am Cancer Soc.

[20] Bacci G, Picci P, Pignatti G (1991). Neoadjuvant chemotherapy for nonmetastatic osteosarcoma of the extremities. Clin Orthop Relat Res.

[21] Hung GY, Yen HJ, Yen CC (2016). Improvement in high-grade osteosarcoma survival: results from 202 patients treated at a single institution in Taiwan. Medicine (Baltimore).

[22] Bielack SS, Kempf-Bielack B, Delling G (2002). Prognostic factors in high-grade osteosarcoma of the extremities or trunk: an analysis of 1,702 patients treated on neoadjuvant cooperative osteosarcoma study group protocols. J Clin Oncol.

[23] Goorin AM, Perezatayde A, Gebhardt M (1987). Weekly high-dose methotrexate and doxorubicin for osteosarcoma: the Dana-Farber Cancer Institute/the Children’s hospital—study III. J Clin Oncol.

[24] Seker MM, Seker A, Aksoy S, Ozdemir N, Uncu D, Zengin N (2014). Clinicopathologic features and prognosis of osteosarcoma in Turkish adults. Asian Pac J Cancer Prev.

