# Supplementary material for: Comparison of clinical features, management and outcomes of osteosarcoma located in proximal fibula and proximal tibia: a propensity score matching analysis
Source: BMC Cancer. 2018 Nov 29;18:1195. doi: 10.1186/s12885-018-5062-6 (PMC6267921; doi:10.1186/s12885-018-5062-6)
Supplement: Supplementary file 1 — Figure S1. The flow diagram for inclusion of patients. This flow diagram shows the process of inclusion of patients. Figure S2. An example of chemotherapy protocol. This picture shows an example of chemotherapy protocol with 9 courses of adjuvant chemotherapy, patients included in our study received 9 or more adjuvant chemotherapy courses. Figure S3. An example of measuring tumor volumes (TVS) and extraosseous tumor volumes (ETVS). A. Measurement of tumor length from coronary section. B. Measurement of tumor width and depth from transverse section. C. Measurement of intraosseous tumor length from coronary section. D. Measurement of intraosseous tumor width and depth from transverse section. (DOCX 531 kb) [file 12885_2018_5062_MOESM1_ESM.docx]

**Figure S1. The flow diagram for inclusion of patients.**

Osteosarcoma from January 2000 to February 2015

（N=1133）

**Figure S2**. An example of chemotherapy protocol

Neo-adjuvant Chemotherapy

**MTX**

**MTX**

**DDP**

**ADM**

**IFO、**

**Surgery**

**1**

**2**

**3**

**4**

Adjuvant Chemotherapy

**MTX**

**DDP**

**ADM**

**IFO、**

**1**

**2**

**3**

**4**

**MTX**

**DDP**

**ADM**

**IFO、**

**MTX**

**DDP**

**ADM**

**IFO、**

**5**

**6**

**7**

**8**

**9**

**Figure S3.** An example of measuring tumor volumes (TVS) and extraosseous tumor volumes (ETVS).


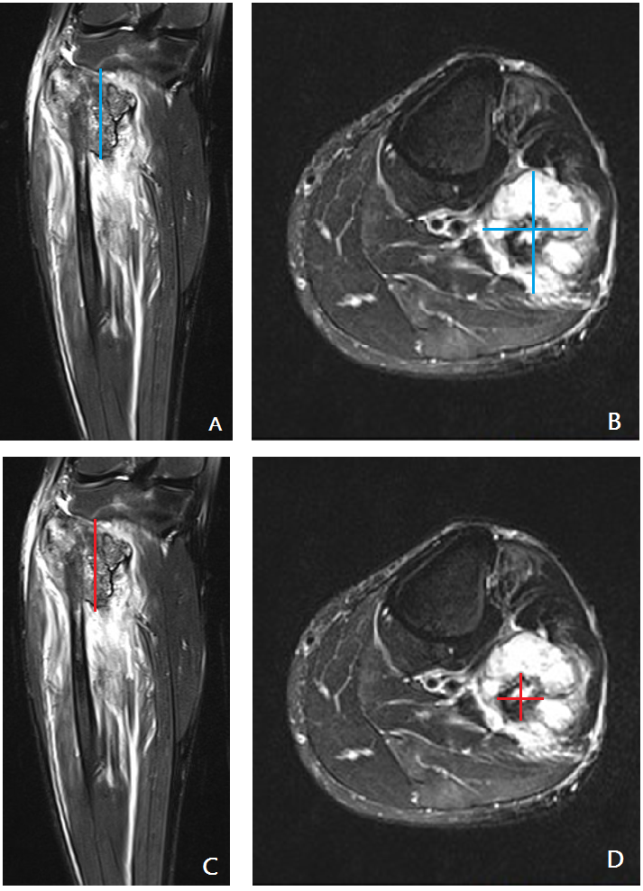


A. Measurement of tumor length from coronary section. B. Measurement of tumor width and depth from transverse section. C. Measurement of intraosseous tumor length from coronary section. D. Measurement of intraosseous tumor width and depth from transverse section.
